# Complete Sequence of the Closed Circular Extrachromosomal Element of Naegleria jadini Willaert and Ray (Strain ITMAP400)

**DOI:** 10.1128/mra.00061-23

**Published:** 2023-03-30

**Authors:** Brian T. Nguyen, Nora M. Chapman, Holly A. F. Stessman, Steven Tracy, Kristen M. Drescher

**Affiliations:** a Department of Medical Microbiology and Immunology, Creighton University, Omaha, Nebraska, USA; b Department of Pathology and Microbiology, University of Nebraska Medical Center, Omaha, Nebraska, USA; c Department of Pharmacology and Neuroscience, Creighton University, Omaha, Nebraska, USA; University of California, Riverside

## Abstract

Amoebae of the *Naegleria* genus carry all ribosome-encoding DNA on closed circular extrachromosomal elements (CERE). We report the sequence of the CERE of Naegleria jadini (strain Willaert and Ray).

## ANNOUNCEMENT

Ribosomal DNA (rDNA) of the *Naegleria* genus is encoded on a closed circular extrachromosomal element (CERE) (1) in the nucleolus (2–6). Each trophozoite has approximately 4,000 copies of the CERE (2, 3), which are postulated to replicate independently (2). A single origin of DNA replication (7) was mapped in the Naegleria gruberi CERE nonribosomal sequences (NRS) (6). The NRS are poorly conserved between species (1). N. jadini was isolated in Antwerp, Belgium, in 1971 (8) and is taxonomically related to N. fowleri (9, 10) but antigenically distinct (11). *N. jadini* was obtained from the ATCC.

*N. jadini* trophozoites were cultured in modified PYNFH medium at 25°C. Confluent flasks were chilled for 5 min, and then detached trophozoites were harvested. *N. jadini* CERE were isolated using a plasmid minikit (Qiagen). Supercoiled CERE DNA was isolated from 0.8% agarose gels and purified using the Monarch DNA gel extraction kit (New England Biolabs); as previously determined *Naegleria* CERE sequences have numerous repeat regions, shearing of the DNA was not desired. CERE DNA was digested with BspEI (New England Biolabs), yielding a linear product of approximately 11 to 12 kbp as determined by agarose gel electrophoresis. Further processing and sequencing were performed at the Roy J. Carver Biotechnology Center at the University of Illinois—Urbana Champaign. DNA quality and quantification were determined using the Agilent Femto Pulse system (Agilent). The library was prepared using the PacBio SMRTbell express template prep kit 3.0 (Pacific Biosciences), and sequencing was performed on a PacBio Sequel IIe using the circular consensus sequencing (CCS) sequencing mode and 30-h movies.

As mitochondrial DNA restriction fragments were coisolated in this procedure, a BLAST alignment identified mitochondrial sequences from the resultant sequence files. Sequences with ≥95% mitochondrial identity to the mitochondrial genome of *N. gruberi* (GenBank accession number NC_002573.1) were removed from the analysis (E value, 1e-50; remaining settings “default”), filtering out 891/72,572 (1.2%) reads. Due to the homology of the rDNA between *Naegleria* species, a BLAST alignment was performed on the remaining 71,681 reads to identify homology with *N. gruberi* rDNA (GenBank accession number AB298288). The BLAST parameters were ≥95% homology to the rDNA sequence, E value of 1e−50, and the remaining parameters default. A total of 51,695/71,681 (72.0%) read sequences were identified as having rDNA identity. The remaining 19,986/71,681 (28.0%) were removed from analysis. Reads were assembled through SPADES Assembler 3.15.5 into contigs and a consensus. Options were “--isolate” and “—plasmid—only-assembler.” The remaining parameters were default. The total number of reads over 10 kbp was 5,848. Characteristics of the CERE are provided in Table 1.

**TABLE 1:** CERE characteristics of *N. jadini* Willaert and Ray

Characteristic	Value
Genome size (bp)	11,825
GC content (%)	42.1
No. of contigs	51
BLAST coverage to *N. gruberi* rDNA (%)	50
Avg read length (bp)^*a*^	3,635.9
Total no. of reads^*a*^	51,695
*N*_50_	10,186
GenBank accession no.	OP997288
SRA accession no. (PacBio)	SRR22270901
BioProject no.	PRJNA887419
BioSample no.	SAMN31166777
SRA accession no. (Plasmidsaurus)	SRR23509813

a Values represent figures after BLAST alignment against mitochondrial and *N. gruberi* rDNA as discussed in the text. When sequences were further filtered to identify sequences between 10 and 13 kbp, the average read length was 11,665 bp and total reads were 5,848.

To test that a complete assembly consensus was obtained, uncut CERE DNA was sequenced with Oxford Nanopore technology (https://www.plasmidsaurus.com/), using the V10 chemistry library prep kit with the R9.4.1 flow cell. The base call software was Guppy. A Clustal alignment of the Plasmidsaurus sequences and the assembled consensus was performed to verify that there was no insertion at the BspEI restriction site in the 18S rDNA sequenceBandage, using two sequencing platforms, and long reads (10 to 13 kbp) were used to resolve repeat regions and ambiguities to complete the assembly consensus.

### Data availability.

The consensus sequence has been deposited in GenBank under accession number OP997288. The first nucleotide of the 18S rDNA subunit has been designated position 1 in the sequence. The PacBio raw reads have been deposited in the NCBI Sequence Read Archive (SRA) and can be found under accession number SRR22270901. The BioProject and BioSample are deposited under accession numbers PRJNA887419 and SAMN31166777, respectively. The Plasmidsaurus raw reads are deposited under accession number SRR23509813.
